# Mid-infrared InAs/InP quantum-dot lasers

**DOI:** 10.1038/s41377-025-02167-4

**Published:** 2026-01-12

**Authors:** Yangqian Wang, Hui Jia, Jae-Seong Park, Haotian Zeng, Igor P. Marko, Matthew Bentley, Khalil El Hajraoui, Shangfeng Liu, Bo Yang, Calum Dear, Mengxun Bai, Huiwen Deng, Chong Chen, Jiajing Yuan, Jun Li, Kongming Liu, Dominic A. Duffy, Zhao Yan, Zihao Wang, Stephen J. Sweeney, Qiandong Zhuang, Quentin M. Ramasse, Siming Chen, Mingchu Tang, Qiang Li, Alwyn Seeds, Huiyun Liu

**Affiliations:** 1 Department of Electronic and Electrical Engineering, University College London, London, UK; 2 James Watt School of Engineering, University of Glasgow, Glasgow, UK; 3 Physics Department, Lancaster University, Lancaster, UK; 4 SuperSTEM, SciTech Daresbury Science and Innovation Campus, Daresbury, UK; 5 York NanoCentre & Department of Physics, University of York, York, UK; 6 School of Physics and Astronomy, Cardiff University, Cardiff, UK; 7 Institute of Physics, Chinese Academy of Sciences, Beijing, China; 8 School of Chemical and Process Engineering and School of Physics and Astronomy, University of Leeds, Leeds, UK

**Keywords:** Semiconductor lasers, Quantum dots, Mid-infrared photonics

## Abstract

Mid-infrared semiconductor lasers operating in the 2.0–5.0 μm spectral range play an important role for various applications, including trace-gas detection, biomedical analysis, and free-space optical communication. InP-based quantum-well (QW) and quantum-dash (Qdash) lasers are promising alternatives to conventional GaSb-based QW lasers because of their lower cost and mature fabrication infrastructure. However, they suffer from high threshold current density (*J*_th_) and limited operation temperatures. InAs/InP quantum-dot (QD) lasers theoretically offer lower *J*_th_ owing to their three-dimensional carrier confinement. Nevertheless, achieving high-density, uniform InAs/InP QDs with sufficient gain for lasing over 2 μm remains a major challenge. Here, we report the first demonstration of mid-infrared InAs/InP QD lasers emitting beyond 2 μm. Five-stack InAs/In_0.532_Ga_0.468_As/InP QDs grown by molecular-beam epitaxy exhibit room-temperature photoluminescence at 2.04 μm. Edge-emitting lasers achieve lasing at 2.018 μm with a low *J*_th_ of 589 A cm^−2^ and a maximum operation temperature of 50 °C. Notably, the *J*_th_ per layer (118 A cm^−2^) is the lowest ever reported for room-temperature InP-based mid-infrared lasers, outperforming QW/Qdash counterparts. These results pave the way for a new class of low-cost, high-performance mid-infrared light sources using InAs/InP QDs, marking a notable step forward in the development of mid-infrared semiconductor lasers.

## Abstract (web summary)

Mid-infrared 2 μm InAs/InP quantum-dot lasers is first demonstrated, with a low threshold current density of 118 A cm^−2^ per layer and a maximum operating temperature of 50 °C.

## Graphical abstract

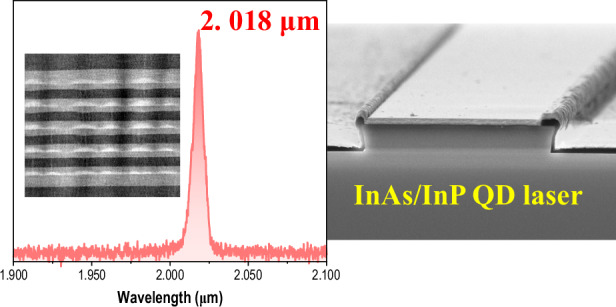

## Introduction

Mid-infrared semiconductor lasers operating in the 2.0–5.0 µm window have attracted significant interest for applications including trace-gas detection, molecular spectroscopy, free-space optical communication, and medical diagnostics^1,2^. While GaSb-based quantum-well (QW) lasers have dominated this regime, their relatively high production cost, low thermal conductivity, and incompatibility with standard photonic platforms hinder their widespread adoption^3–7^. InP-based QW lasers, leveraging lower cost and mature manufacturing infrastructures, have emerged as promising Sb-free alternatives for achieving 2–2.5 µm emission^7–9^. Notable progress has been achieved through various cavity designs, including triangular QW, distributed feedback, and type-II lasers^8,10,11^. For instance, Gu et al.^11^ demonstrated 2.37 µm InP-based edge-emitting lasers using triangular QWs, achieving a room-temperature (RT) *J*_th_ of 1.3 kA cm^−2^ and a maximum operating temperature of 65 °C. In addition, Sprengel et al.^8^ extended the emission range to 2.2–2.6 μm with type-II structures, albeit with higher *J*_th_ values (1.8–4.0 kA cm^−2^ at 2.2 μm). However, compared to state-of-the-art GaSb-based QW lasers (*J*_th_ < 100 A cm^−2^ at ~2.0–2.1 μm)^12^, mid-infrared InP-based QW lasers still exhibit substantially higher *J*_th_ and restricted operating temperatures, highlighting the need for alternatives with improved operation performance.

Self-assembled InAs quantum-dot (QD) lasers, with three-dimensional carrier confinement, offer several advantages over conventional QW lasers, including low *J*_th_, robust tolerance to defects, potential for temperature-insensitive operation, and low linewidth enhancement factor—features critical for mid-infrared applications^13–18^. Indeed, InAs/GaAs QD lasers at 1.3 µm and InAs/InP QD lasers at 1.55 µm for optical communications have demonstrated static and dynamic laser performances comparable, or even exceeding, to those of mainstream QW counterparts in certain aspects, while also showing the promise for monolithic integration into Si-based platforms^19–22^. Although implementing InAs/InP QD gain medium into mid-infrared light source has substantial potential benefits, extending the emission wavelength beyond 2 µm remains an unresolved challenge. A major limitation is the weak strain energy for InAs QD formation, caused by a small lattice mismatch (~3.2%) between InAs and InP, which makes it difficult to control the dot height and size, thereby resulting in significant size inhomogeneity^23,24^. Compared to 1.55 µm InAs/InP QDs, achieving 2 µm emission requires larger dot volumes, which exacerbates size inhomogeneity and increases the likelihood of generating defective dots that exceed the elastic strain relaxation limit. In addition, InAs quantum-dashes (Qdash) elongated along the $[1\bar{1}0]$ direction are preferentially formed on (001) InP substrate due to anisotropic diffusion of indium adatoms, further complicating the growth of round-shaped QDs^25^. Consequently, the size/shape inhomogeneity and low density of InAs/InP QDs lead to insufficient optical gain, posing critical obstacles to realize mid-infrared InAs/InP QD lasers.

To overcome these hurdles, various growth strategies have been explored, including modifications of underlying layers of InAs QDs and adjustments to the QD nucleation process^26–29^. For example, Qiu et al.^26^ found that incorporating a thin GaAs interface layer between the InAs QD layer and the underlying InGaAs layer during QD growth effectively suppressed indium adatom migration, resulting in a more controlled QD formation process. With this method, a high QD density of ~3 × 10^10 ^cm^−2^ and enhanced RT photoluminescence (PL) intensity at around 2 µm were achieved. Additionally, Tang et al.^27^ utilized a two-step growth method, namely fast InAs nucleation followed by atomic layer epitaxy, to form InAs QDs on In_x_Ga_1-x_As/InP matrices and achieved QDs exhibiting 2.35 μm PL at 77 K, with a dot density of ~1.1 × 10^10 ^cm^−2^ and a narrow PL full-width at half-maximum (FWHM) of 25.5 meV.

Despite these efforts, 2 µm emission in InAs/InP QDs remains confined to PL emission, with no prior demonstration of lasing at this wavelength. Moreover, existing studies rely on single-layer QD structures, which inherently lack the material gain for lasing due to their limited areal density. While multi-stack QD structures could amplify optical gain, these architectures introduce formidable additional challenges, such as strain accumulation and interlayer strain coupling, complicating epitaxial growth. These unresolved challenges underscore a stark technological gap: while InP-based QW and Qdash lasers have achieved 2 µm operation, the unique advantages of InAs/InP QDs—low *J*_th_ and temperature-insensitive operation—remain untapped for the mid-infrared applications. Therefore, achieving 2 µm emission in multi-stacked, high-density, and uniform InAs/InP QDs is of critical importance to unlock the potential of 2 µm InAs/InP QD lasers for next-generation mid-infrared light sources.

Here, we demonstrate for the first time five-stack InAs/In_0.532_Ga_0.468_As/InP QD lasers on n-type (001) InP substrates grown by molecular beam epitaxy (MBE), achieving RT lasing at 2.018 µm. By precisely manipulating the growth conditions to suppress the anisotropic diffusion of indium atoms, round-shaped and uniform InAs/InP QDs with a dot density of 1.83 × 10^10 ^cm^−2^ and a PL FWHM of 42.4 meV at RT were obtained. High-resolution scanning transmission electron microscopy (STEM) images from both $[110]$ and $[1\bar{1}0]$ directions confirmed the dot morphology. The as-cleaved InAs/InP QD edge-emitting lasers under pulsed injection exhibited a *J*_th_ of 589 A cm^−2^, corresponding to the *J*_th_ per layer of 118 A cm^−2^, with a maximum operating temperature of 50 °C. To the best of our knowledge, the *J*_th_ per layer achieved here is the lowest value, surpassing the reported RT InP-based QW and Qdash lasers until now.

## Results

### Optimization of InAs/InP QDs

To obtain InAs QDs emitting beyond 2 µm, 20 nm In_0.532_Ga_0.468_As layer lattice-matched to InP was employed to sandwich the InAs QDs, thereby extending wavelength through a reduced band offset. In addition, growth parameters to suppress the anisotropic diffusion of indium were selected at the initial stage of the growth. These include a high InAs growth rate of 0.42 monolayer (ML) s−^1^, the use of As_2_ instead of As_4_ for the QD growth, and a relatively low QD growth temperature regime between 480 and 510 °C. On InP (001) substrate surface, the anisotropy of the indium diffusion coefficient along $[110]$ and $[1\bar{1}0]$ directions at typical growth temperature (~500 °C) is known to be a factor of ~3 due to the different number of lateral bonds the group III atom forms along the two directions^30^. Therefore, elongated structures could be expected if the diffusion process is not controlled. A high In deposition rate with a relatively low growth temperature can limit the surface migration of the adatoms as the large amount of adatoms will have less energy to move on the surface^31^. Using As_2_ eliminates the process of cracking As_4_ on the surface, providing stable As-terminated atomic steps along the $[1\bar{1}0]$ direction and thus significantly mitigating indium adatom migration anisotropy^32^. Based on these initial growth conditions for QD formation, the InAs deposition thickness, V/III ratio, and growth temperature were optimized to realize InAs/InP QDs emitting beyond 2 µm.

**Fig. 1:**
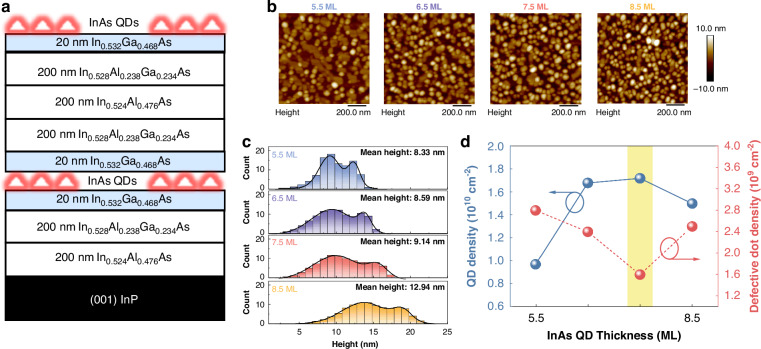
Epitaxial structure and morphological characterization of single-layer InAs/InP QDs with various InAs thickness. **a** Schematic epitaxy structure of single-layer InAs/InP QDs sandwiched by In_0.532_Ga_0.468_As barrier layers. **b** AFM images of uncapped InAs QDs for various InAs deposition thicknesses from 5.5 to 8.5 ML. **c** Histograms of InAs QD height from 5.5 to 8.5 ML, where bimodal distribution is minimized at 7.5 ML. **d** Round-shaped and defective (2-D feature and/or coalesced) dot densities as a function of InAs thickness

Figure 1a displays a schematic illustration of single-layer InAs/InP QD epitaxial structure used to optimize QD growth conditions. First, 5.5, 6.5, 7.5, and 8.5 MLs of InAs were deposited to determine the optimal thickness for high-density, uniform QDs at a growth temperature of 485 °C and a V/III ratio of 18. Figure 1b shows 1 × 1 µm^2^ atomic force microscopy (AFM) scans of as-grown InAs/InP QDs. A low density (9.70 × 10^9 ^cm^−^^2^) of round-shaped QDs was initially formed at 5.5 ML with the presence of large two-dimensional (2-D) features, which is an indication of the incomplete formation of QDs with insufficient strain accumulation. For the weakly-strained InAs/InP material system, the total strain energy can be partially relaxed by the formation of an interfacial alloy between the InAs and the InGaAs layer. The new interfacial InGaAs alloy introduces less strain than InAs would have done, further aggravating the formation of high-density, round-shaped QDs^33^. On the other hand, increasing InAs thickness to 6.5 and 7.5 MLs yielded significantly improved dot densities of 1.68 × 10^10 ^cm^−2^ and 1.72 × 10^10 ^cm^−2^, respectively, and greatly reduced 2-D features. Further increasing the InAs thickness to 8.5 ML, however, led to a decreased dot density of 1.50 × 10^10 ^cm^−2^, accompanied by an increase in the number of coalesced islands and size nonuniformity. Figure 1c presents dot-height histograms extracted from each corresponding AFM image, showing a clear bimodal size distribution that needs to be resolved. The mean height increases from 8.33 nm at 5.5 ML to 12.94 nm at 8.5 ML. The 7.5 ML sample exhibits the minimum bimodal size distribution with a mean height of 9.14 nm and a standard deviation of 3.32 nm. Figure 1d summarizes both round and defective (2-D feature and/or coalesced) dot densities as a function of InAs thickness. The defective dot density decreases with increasing InAs thickness, reaching a minimum of 1.60 × 10^9 ^cm^−2^ at 7.5 ML before rising again to 2.5 × 10^9 ^cm^−2^ at 8.5 ML.

**Fig. 2:**
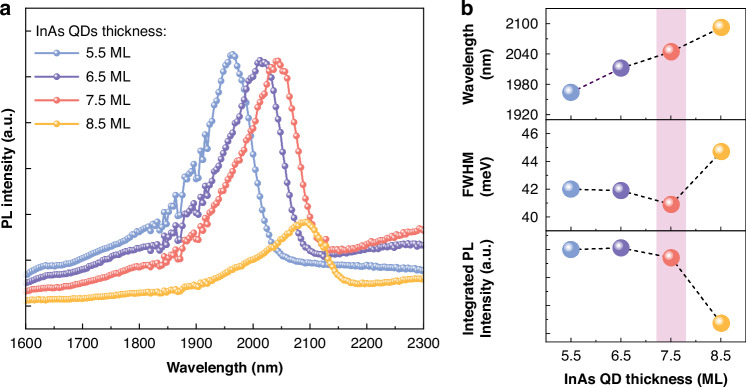
Optical characterization of single-layer InAs/InP QDs with various InAs thickness. **a** PL spectra of single-layer InAs/InP QDs with various InAs thicknesses from 5.5 to 8.5 ML at RT. **b** Summary of central peak emission wavelength, FWHM, and integrated PL intensity of InAs/InP QDs as a function of InAs thickness

For optical characterization of single-layer InAs/InP QDs, PL spectra were measured at RT, as shown in Fig. 2a. As the InAs thickness increases, the emission peak wavelength redshifts with the 6.5, 7.5, and 8.5 ML samples emitting beyond 2 μm (2012, 2044, and 2092 nm, respectively), consistent with the larger dot sizes presented in Fig. 1c. The peak intensities for 5.5, 6.5, and 7.5 ML samples are almost the same while for the 8.5 ML sample, the PL intensity is significantly reduced. Figure 2b summarizes the peak wavelength, FWHM, and integrated intensity of the PL from these structures. The FWHM narrows from 42.0 meV at 5.5 ML to a minimum of 40.9 meV at 7.5 ML, then broadens to 44.7 meV at 8.5 ML, reflecting that 7.5 ML provides the most uniform dot ensemble. The integrated PL intensity for 5.5–7.5 ML remains nearly constant with only small decrease at 7.5 ML before dropping sharply at 8.5 ML, ascribed to increased non-radiative recombination from the accumulated strain-induced crystalline defects^34,35^. These originate from the thick InAs layer at 8.5 ML and In migration from the underlying InGaAs layer, exceeding the elastic relaxation limit, consistent with earlier observations^26,36^.

**Fig. 3:**
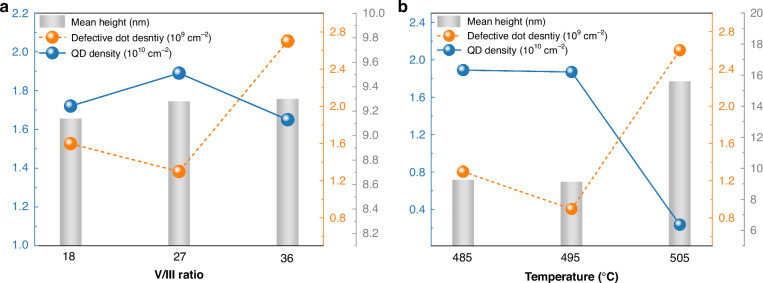
Morphological characteristics of single-layer InAs/InP QDs grown with varied V/III ratio and growth temperature. **a** V/III ratio variation and **b** growth temperature variation. QD density (blue dots) uses left axis; defective dot density (orange dots) and mean height of QDs (gray bars) use right axis

To further optimize the dot morphology, the influence of V/III ratio and QD growth temperature were investigated. Morphological characteristics, including QD density, defective dot density, and QD mean height, of single-layer InAs/InP QDs grown under different V/III ratios of 18, 27, and 36 with optimized 7.5 ML InAs at 485 °C are shown in Fig. 3a. Compared with the sample under V/III ratio of 18 (the previously optimized sample), the dot density further increases to 1.89 × 10^10 ^cm^−2^ for sample under V/III ratio of 27, while the dot morphology remains unchanged as confirmed by 1 × 1 μm^2^ AFM images (Supplementary Fig. S1a). However, further increasing the V/III ratio to 36 results in a decreased dot density of 1.65 × 10^10 ^cm^−2^ and increased 2-D features with a defective dot density of 2.7 × 10^9 ^cm^−2^. The mean heights for all ratios remained constant (9.14–9.30 nm), while the ratio of 27 achieved the most uniform dot distribution, showing no bimodality (Supplementary Fig. S1b). The observed increase in dot density by the elevated V/III ratio of 27 can be explained by the enhanced surface reaction efficiency brought by the abundant As_2_ supply. Simultaneously, the enriched As pressure further restrains the anisotropic surface diffusion of the indium adatoms, promoting the formation of a more uniform dot ensemble. However, out of the optimal V/III ratio window, i.e., 36 in our case, coalesced dots begin to emerge due to reduced adatom mobility. In addition, excess As can lead to deteriorated material and interface quality, degrading the optical properties^37^.

Figure 3b shows morphological properties of InAs/InP QDs grown at 485, 495, and 505 °C with optimized 7.5 ML InAs and V/III ratio of 27. The dot density for 485 and 495 °C remains nearly constant, while the defective dot density slightly decreases at 495 °C from 1.3 × 10^9 ^cm^−2^ to 0.9 × 10^9 ^cm^−2^. In contrast, the dot density sharply decreases to 2.4 × 10^9 ^cm^−2^ at 505 °C, and the defective dot density increases to 2.6 × 10^9 ^cm^−2^. The AFM images further reveal that the dot morphology remains similar for 485 and 495 °C, while for 505 °C flatter and elongated dashes are presented among disparted large, coalesced dots (Supplementary Fig. S2a). This indicates that at this temperature, the reduced sticking coefficient and high adatom mobility promoted anisotropic surface diffusion, resulting in elongated structures. Moreover, the initially formed smaller dots tend to coalesce with adjacent larger ones via a ripening mechanism, ultimately giving rise to large, spatially disparted dots. Additionally, substantial variation in dot height was observed with changes in growth temperature. While the increased dot height (~15.6 nm) at 505 °C facilitates a longer emission wavelength and a deeper confinement potential for carriers^38^, it also reduces the quantum confinement effect, increasing susceptibility to Auger recombination and thermal sensitivity^39^. Furthermore, the broader dot-height distribution at 505 °C (Supplementary Fig. S2b) can induce significant inhomogeneous gain broadening, diminishing peak gain and thereby increasing threshold requirements^24^. In contrast, dots grown at 495 °C with a height of ~9 nm, showing a narrower dot-height distribution without bimodality, can offer sufficient emission redshift while maintaining stronger carrier confinement and a higher optical gain, both crucial for achieving lasing.

**Fig. 4:**
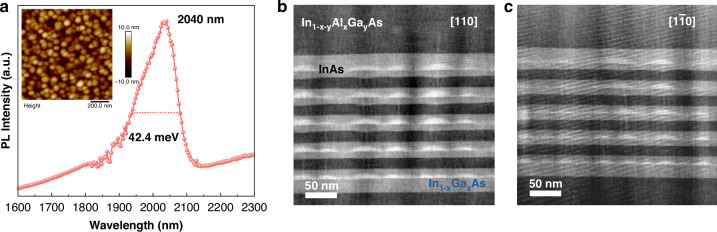
Optical and structural characterization of five-stack InAs/InP QDs. **a** PL spectrum of five-stack InAs/InP QD structure at RT with central emission wavelength of 2040 nm and FWHM of 42.4 meV. The inset presents AFM image of uncapped five-stack InAs/InP QDs. Cross-sectional HAADF images showing QD morphologies in five-stack InAs/InP QD laser structure along **b** $[110]$ and **c** $[1\bar{1}0]$ directions

Based on the optimized conditions (7.5 ML InAs, V/III ratio of 27, and QD growth temperature of 495 °C), two five-stack InAs/InP QD samples were grown: one dedicated to structural/optical characterization and the other fabricated into lasers. Details of both structures are described in Method section. To characterize the five-stack InAs/InP QD structure, PL and AFM were carried out. Figure 4a shows the RT PL emission at 2040 nm with a narrow FWHM of 42.4 meV. The PL characteristics for the five-stack InAs/InP QD structure are similar to the optimized single-layer InAs/InP QD structure. The inset of Fig. 4a exhibits a 1 × 1 µm^2^ AFM scan image for the surface QDs on top of the five-stack InAs/InP QD structure, confirming a dot density of 1.83 × 10^10 ^cm^−2^. The in-depth morphology of QDs in the five-stack InAs/InP QD laser structure was further confirmed by high-angle annular dark field (HAADF) STEM images in both $[110]$ and $[1\bar{1}0]$ directions, as shown in Fig. 4b, c, respectively. Since contrast in HAADF image is proportional to sample thickness and average atomic number Z as I ∼ Z^n^ (*n* = 1.4–1.8)^40^, the bright contrast of the pyramid-shaped island in the InGaAs layers shows the successful formation of InAs QDs in both directions. Bright lines at the tip of the pyramid-shaped QDs and buffer layers can be observed in the HAADF images in both $[110]$ and $[1\bar{1}0]$ directions, indicating In diffusion during the capping and annealing steps. During the growth of the 10 nm InGaAs capping layer above the dots, InGaAs will first deposit between the dots due to the higher strain at the apex of dots^41^. When the surface energy equals, the growth will initiate above the dots with In atoms preferentially growing on top of InAs QDs owing to smaller lattice mismatch compared with the InGaAs matrix^42^ and lower nucleation energy in the relaxed InAs islands^43^, which results in an In-rich region above InAs QDs. The same mechanism follows in the InAlGaAs growth. In the subsequent annealing step, partial desorption of In from the InAlGaAs layer occurs, accompanied by In diffusion from the InAs QDs toward the surface, which is a result of the reduced mobility of In in an InGaAs matrix than in In-rich regions such as areas above InAs QDs^44^. This further enhances the indium-rich regions above the dots, visible as the bright-contrasted lines in HAADF images in Fig. 4b, c. Such In diffusion modifies the local composition and band offsets near the QDs^43,45^, which may reduce effective carrier confinement. From the bottom to the top QD layers, a clear trend of size increase is observed. Specifically, the InAs QDs in each layer from bottom to top have average lengths of 34.5, 41.4, 42.9, 51.2, and 54.2 nm, and average heights of 6.1, 6.4, 6.6, 8.2, and 9.2 nm, respectively, accompanied by a decrease in dot density. The size increase and density decrease of the dot at the higher QD layers can be explained by the strain-coupling effect, originating from the relatively large QD size in the InAs/InP material system^46,47^. The excellent dot symmetry ensures superior carrier confinement and minimizes inhomogeneous broadening, which is crucial for achieving high gain required for lasing.

### Performance of five-stack InAs/InP QD lasers

**Fig. 5:**
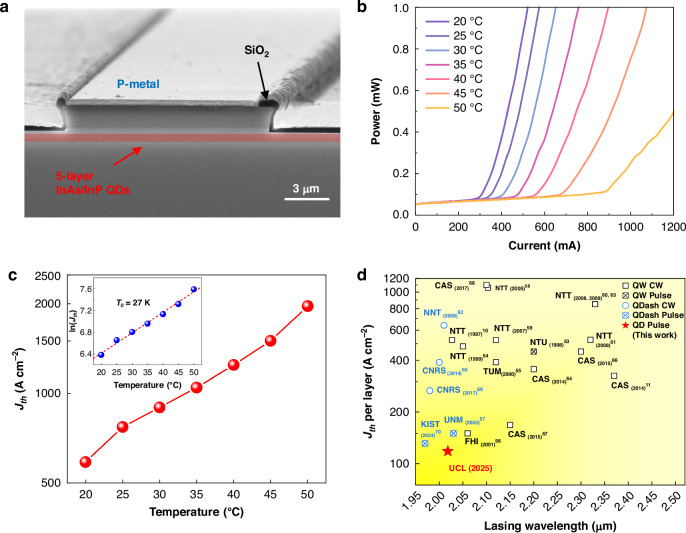
Laser performance characterization. **a** Cross-sectional SEM image of as-cleaved five-stack InAs/InP QD lasers. **b** Temperature-dependent L-I characteristics for the QD laser with a cavity width of 15 μm and a length of 3 mm. **c** The *J*_th_ variation as a function of temperature. The inset displays characteristic temperature, *T*_0_. **d** Comparison of previously reported *J*_th_ per layer of InP-based QW and Qdash lasers emitting in the 2–2.5 μm window

Figure 5a illustrates a cross-sectional scanning electron microscopy (SEM) image of edge-emitting laser fabricated from the optimized five-stack InAs/InP QDs. The fabricated lasers were characterized under pulsed injection (1% duty cycle, 1 μs pulse width) to minimize self-heating effects. Figure 5b exhibits temperature-dependent light-current (L-I) curves for InAs/InP QD lasers with a cavity width of 15 μm and a length of 3 mm. The device achieved a low *J*_th_ of 589 A cm^−^^2^ at RT, corresponding to the *J*_th_ per layer of 118 A cm^−2^, and a maximum operating temperature of 50 °C. The maximum power of this device at RT was measured to be 8.5 mW per facet at an injection current of 1250 mA (Supplementary Fig. S3a). Figure 5c depicts a graph showing the *J*_th_ variation as a function of temperature in a logarithmic scale. An increase in *J*_th_, up to 1.96 kA cm^−2^ at 50 °C, was observed. The characteristic temperature (*T*_0_), a measure of temperature dependence of *J*_th_, was calculated as 27 K (the inset of Fig. 5c). Note that the temperature sensitivity remains relatively high, arising from both fundamental physical limits at the 2 μm wavelength and process-induced degradation. Compared to conventional C-/L-band InAs/InP QD lasers, the lower *T*_0_ stems from fundamental challenges inherent to long-wavelength operation. The large dot size required for 2 μm emission reduces quantum confinement effect and the associated transition energy, substantially enhancing the Auger recombination rate and increasing thermal sensitivity^48^, while the free carrier absorption that scales with *λ*^2^ becomes a more significant internal loss mechanism at this wavelength^49^. These effects collectively impose a fundamental limit on the thermal stability of mid-infrared QD lasers. However, the *T*_0_ of 27 K is lower than typical values reported for InP-based QW/QDash lasers (often in the 35–50 K range). This further thermal degradation is attributed to process-induced factors in present device. The heterogeneous regrowth of the p-InP cladding layer introduces defects that act as efficient Shockley–Read–Hall recombination centers^50^, while the broad, shallow ridge waveguide formed by chemical wet etching contributes to the undesirable current spreading and higher-order mode competition, increasing the threshold current (*I*_th_) and thus heat generation^51^. The interplay between these fundamental limits and extrinsic fabrication issues currently obscures the temperature-insensitive behavior expected from QDs.

The RT L-I characteristics for the device with a cavity width of 15 μm and different cavity lengths from 1 to 3 mm were also measured (Supplementary Fig. S3b). The *J*_th_ for the 1, 1.5, and 2 mm length devices were measured to be 993, 760, and 664 A cm^−2^, respectively. Based on the *J*_th_ values from different cavity lengths, inverse cavity length versus *J*_th_ was plotted, from which the transparency current density (*J*_tr_) of 367.7 A cm^−2^, corresponding to the *J*_tr_ per layer of 73.5 A cm^−2^, was extracted (Supplementary Fig. S3c). To further evaluate the device performance, key laser parameters were extracted from cavity-length-dependent measurements. Internal loss (*α*_i_) and internal quantum efficiency (*η*_i_) were extracted from the dependence of the external differential quantum efficiency (*η*_d_) on cavity length (Supplementary Fig. S3d). A linear fit to this data yielded an *α*_i_ of ~6.7 cm^−1^ and an *η*_i_ of 60%. Furthermore, the material differential gain (*dg/dn*) was estimated to be 8.05 × 10^−15 ^cm^2^, where confinement factor (*Γ* = 0.03), QD fill factor (*ξ* = 0.51), radiative lifetime (*τ*_r_ = 1 ns)^52^ and the experimentally calculated values including active region thickness (*d* = 250 nm), *η*_i_ = 60%, and *α*_i_ = 6.7 cm^−1^ were used.

Figure 5d summarizes RT *J*_th_ per layer—a key parameter for semiconductor lasers—of mid-infrared InP-based QW and Qdash lasers emitting in the 2–2.5 μm window, as reported by various groups over the past two decades^10,11,53–70^. Despite substantial efforts, the *J*_th_ per layer of QW and Qdash lasers has shown little improvement since the first demonstration. In contrast, the first mid-infrared InP-based QD laser demonstrated here achieves a low *J*_th_ per layer, surpassing previously reported values for its QW and Qdash counterparts and demonstrating the advantage of InAs/InP QDs as a gain medium for mid-infrared semiconductor lasers. In addition to a low *J*_*th*_, the inhomogeneously broadened gain spectrum, inferred from the RT PL FWHM of ~42 meV (Fig. 4a), indicates the potential for a wider gain bandwidth than that of typical InP-based QW lasers. This intrinsic property is highly advantageous for applications requiring wavelength tuning or the generation of short optical pulses. Furthermore, while the *T*_0_ in this initial device is currently limited to 27 K by extrinsic factors, the fundamental carrier confinement in QDs provides a pathway to the superior temperature stability witnessed in mature near-infrared QD lasers^71,72^. Although direct lifetime comparisons at 2 μm are not yet available in the literature, the suppressed carrier diffusion and enhanced defect tolerance in QD system suggest a potential for improved reliability comparable to QW/Qdash devices^73^. Therefore, while the low *J*_th_ per layer demonstrated here is a critical milestone, the QD gain medium offers a promising platform for achieving mid-infrared semiconductor lasers with superior efficiency, thermal stability, and gain bandwidth.

**Fig. 6:**
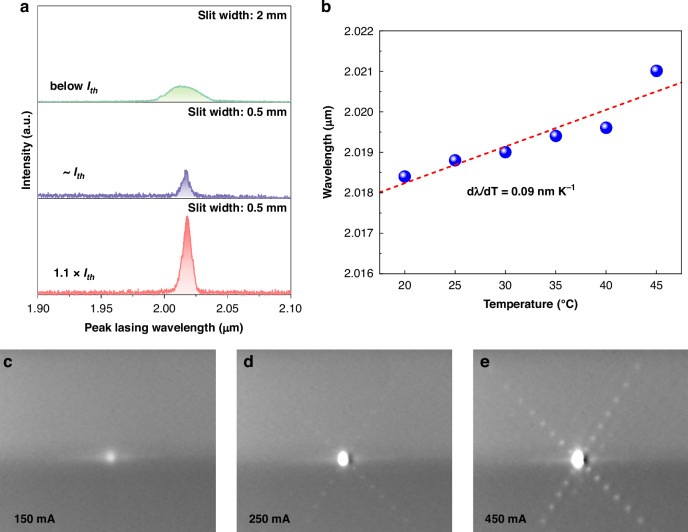
Spectral and spatial emission characteristics. **a** RT lasing spectra of five-stack InAs/InP QD lasers (15 × 3000 µm^2^) at injection currents of 260 mA (below *I*_th_), 265 mA (~*I*_th_), and 290 mA (1.1 × *I*_th_). **b** Temperature-dependent lasing wavelength shift at an injection current of 1.1 × *I*_th_. Far-field patterns obtained from the 8 × 3000 µm^2^ device at injection currents of **c** 150 mA (below *I*_th_), **d** 250 mA, and **e** 450 mA

To characterize the impact of temperature on the lasing wavelength, the temperature-induced wavelength shift was investigated. Figure 6a displays the lasing emission spectra at RT under different current injections. A broad spontaneous emission centered at 2013 nm with a FWHM of 28.6 nm is observed at an injection current of 260 mA (below *I*_th_). At an injection current of 265 mA close to *I*_th_, the peak intensity at 2017 nm arises sharply and FWHM narrows to 6.4 nm, providing evidence of lasing. Further increasing the injection current to 290 mA (1.1 × *I*_th_), the intensity is enhanced with a lasing peak wavelength at 2018.4 nm and a FWHM of 7.4 nm. Note that a wider slit width (2 mm) was used for spontaneous emission measurements than for lasing measurement (0.5 mm) to enhance the collection of weak signals, resulting in strong spontaneous emission intensity below *I*_th_. The observed broad, multimode lasing spectrum is a characteristic of Fabry–Pérot lasers with a long cavity and a broad gain medium. First, the long cavity length (3 mm) results in a very narrow free spectral range. At a lasing wavelength of 2018 nm, the longitudinal mode spacing is calculated to be only ~0.18 nm. This dense mode spectrum allows many longitudinal modes to oscillate simultaneously within the wide, inhomogeneously broadened gain spectrum of the QD ensemble. Second, the shallow, wet-etched wide ridge waveguide with its non-vertical sidewalls provides weak lateral optical confinement, permitting the excitation of higher-order transverse modes. The superposition of these multiple lateral and longitudinal modes results in the broad spectral output observed. Figure 6b presents the temperature-dependent peak lasing wavelength shift, measured at an injection current of 1.1 × *I*_th_. The lasing peak redshifts as the temperature increases, with a small wavelength shift of 0.09 ± 0.018 nm K^−1^. Note that the measurement of lasing peak at 50 °C was constrained by the resolution of the measurement system. To further confirm the spatial coherence of lasing, far-field diffraction patterns were measured from an 8 × 3000 μm^2^ device with improved beam quality, whose L-I characteristic confirming an *I*_th_ of ~200 mA is provided in Supplementary Fig. S4a. Below the *I*_th_ (at 150 mA), the far-field pattern exhibited only spontaneous emission without interference fringes (Fig. 6c). Above the *I*_th_ (at 250 mA), distinct interference fringes emerged, indicating the onset of coherent lasing oscillation (Fig. 6d). At a higher injection current of 450 mA, the pattern showed a bright central lasing spot with well-defined interference features (Fig. 6e).

Looking forward, 2 µm InAs/InP QD laser performance can be enhanced through optimizations in the device architecture, addressing the interrelated challenges of thermal stability, output power, and spectral control. The current limitations in output power (8.5 mW/facet) and thermal sensitivity (*T*_0_ = 27 K) stem primarily from thermal effects and internal losses exacerbated by the present fabrication process. Critical improvements include: (1) monolithic growth process to eliminate the defective regrowth interface, thereby reducing non-radiative recombination; (2) deep-etched, narrow ridge waveguides for enhanced lateral optical confinement and reduced current spreading; and (3) optimized separate confinement heterostructures minimizing the loss arising from free carrier absorption at 2 µm. For practical applications requiring single-mode operation and wavelength stability, the implementation of distributed feedback or distributed Bragg reflector gratings is essential. This comprehensive approach—encompassing material quality, waveguide design, and cavity engineering—provides a clear pathway toward high-power, efficient, continuous-wave operation at RT, which is crucial for unlocking the full potential of InAs/InP QD lasers for mid-infrared applications.

## Discussion

In this work, we demonstrate the first InAs/InP QD lasers operating beyond 2 μm. The round-shaped dot density of 1.83 × 10^10 ^cm^−2^ with a narrow PL FWHM of 42.4 meV at RT was achieved for five-stack InAs/In_0.53_Ga_0.47_As/InP QDs by optimizing the growth conditions. The successful growth of the desired QDs without elongated structure has been confirmed by HAADF STEM imaging from both $[110]$ and $[1\bar{1}0]$ directions. The fabricated five-stack InAs/InP QD lasers (15 μm × 3 mm) exhibited RT lasing at 2.018 μm with a low *J*_th_ of 589 A cm^−2^ under pulsed injection, achieving a maximum operating temperature of 50 °C and a temperature-dependent wavelength shift of 0.09 nm K^−1^. We achieved a record-low *J*_th_ per layer (118 A cm^−2^), outperforming all prior RT InP-based mid-infrared QW and Qdash lasers. These findings not only demonstrate the viability of InAs/InP QDs as a gain medium for mid-infrared 2 μm emission but also represent a significant advance toward low-cost, high-performance 2–2.5 μm light sources for mid-infrared applications.

## Methods

### Growth of five-stack InAs/InP QD structure

The InAs/InP QDs were grown on an n-type (001) InP substrate using the Veeco GEN 930 MBE equipped with a valved arsenic cracker source. Prior to growth, the InP substrate was degassed at 400 °C in the preparation chamber of the MBE facility for 1 h, followed by thermal deoxidation at 500 °C for 1 min under As_2_ overpressure protection. Then, 200 nm In_0.524_Al_0.476_As and 200 nm In_0.528_Al_0.238_Ga_0.234_As layers lattice-matched to InP were deposited at 510 and 495 °C, respectively. The V/III ratio used was 30 for both. A five-stacked QD structure was then deposited. First, 20 nm In_0.532_Ga_0.468_As was grown at 495 °C and then the InAs QDs were grown with optimized conditions described in section 2.1. The QDs were then capped with 10 nm In_0.532_Ga_0.468_As and 15 nm In_0.528_Al_0.238_Ga_0.234_As spacer layer. The substrate temperature was elevated to 525 °C for 3 min to remove point defects and improve material quality. Subsequently, the substrate was cooled to 495 °C to grow the 10 nm In_0.532_Ga_0.468_As. The QD growth was then repeated. The structure was finally completed with another 200 nm In_0.528_Al_0.238_Ga_0.234_As and 200 nm In_0.524_Al_0.476_As. Note that for characterization of surface dot morphology, 200 nm In_0.528_Al_0.238_Ga_0.234_As, 20 nm In_0.532_Ga_0.468_As, and uncapped InAs QDs were also grown on top of the final structure.

### Growth of five-stack InAs/InP QD laser structure

Unless stated otherwise, the growth parameters and procedures of MBE-grown laser structure are same as the InAs/InP QDs, except that Si and Be were used as n-type and p-type dopants, respectively. First, the bottom n-In_0.524_Al_0.476_As and n-In_0.528_Al_0.238_Ga_0.234_As layers with doping density of 5 × 10^18 ^cm^−3^ and 2 × 10^18 ^cm^−3^, respectively, were grown on n-InP substrate, followed by the five-stack InAs/InP QD active region. Subsequently, the upper p-In_0.528_Al_0.238_Ga_0.234_As and p-In_0.524_Al_0.476_As layers with doping concentration of 2 × 10^18 ^cm^−3^ and 5 × 10^18 ^cm^−3^, respectively, were grown. A 10 nm Be-doped In_0.532_Ga_0.468_As layer was deposited, serving as a protection layer to alleviate oxidation during transfer to metal-organic chemical vapor deposition (MOCVD). 1700 nm Zn-doped InP (1 × 10^18 ^cm^−3^) and 200 nm Zn-doped In_0.53_Ga_0.47_As (2 × 10^19 ^cm^−3^) were then deposited by MOCVD as cladding and p-type contact layers, respectively.

### Fabrication of InAs/InP QD lasers

The five-stack InAs/InP QD Fabry–Pérot edge-emitting lasers were fabricated with a ridge width of 15 μm. The ridges were defined using conventional photolithography and wet chemical etching (HCl: H_3_PO_4_ = 1: 3). Subsequently, a passivation layer of 400 nm SiO_2_ was deposited using plasma-enhanced chemical vapor deposition. After opening a window via reactive ion etcher, a p-type metallization of Ti/Au (20/300 nm) was deposited on the exposed top ridge by a sputtering system. The substrate was thinned to 150 μm. For an n-type metallization, Ni/AuGe/Ni/Au (10/150/10/200 nm) layers were deposited on the backside of the sample using a thermal evaporator. To form an Ohmic contact, the samples were annealed at 380 °C for 1 min. The laser bars were cleaved into different cavity lengths without facet coatings.

### Measurement and characterization

Surface morphological characterization of the as-grown InAs QDs were carried out using AFM operated in non-destructive tapping mode. It is equipped with a sharp tip with an apex radius of 20 nm, mounted on a cantilever that oscillates vertically around its resonant frequency (146–236 kHz) as it approaches the sample surface. A photodiode detector monitors variations in the reflection of a laser beam directed at the cantilever, enabling the reconstruction of the nanostructure topography. High-resolution HAADF STEM analysis of the laser samples along two directions of $[110]$ and $[1\bar{1}0]$ was carried out using a Nion UltraSTEM100 dedicated aberration-corrected STEM, operated at 100 kV acceleration voltage, equipped with a cold-field-emission electron gun. The microscope was configured to form a 0.9 nm diameter probe on the sample with a convergence semi-angle of 30 mrad and a probe current of ~30 pA. The angular range of the HAADF detector was calibrated as 90–185 mrad, and images were acquired as series to eliminate stage drift and scanning distortions using rigid and non-rigid registration methods^74^. Thin lamellae were extracted from the same chip along the $[110]$ and $[1\bar{1}0]$ directions, using conventional focused-ion-beam sample preparation methodologies on a Hitachi Ethos NX50000 focused-ion-beam/scanning electron microscope microfabrication platform. After initial lamella extraction using a 30 kV Ga-ion beam, progressively lower Ga^+^ ion acceleration voltages, down to 2 kV, were used to thin the samples to electron transparency.

RT PL spectra of the as-grown samples were measured using a 532 nm laser with a power density of 20 W cm^−2^. The emitted signal was collected and focused using a set of lenses and then coupled into a SPEX 1000 M spectrometer. The optical signal was subsequently detected by an extended InGaAs detector (up to 2.4 μm) connected to a lock-in amplifier. The L-I characteristics of the as-cleaved lasers were mounted on a thermoelectric temperature-controlled stage. The laser devices were measured under pulsed current injection (1 μs pulse width, 1% duty cycle), and the output power signal was collected using Thorlabs PM5020 power meter set as the center wavelength of 2 μm.

The lasing spectra were obtained in free space using a monochromator (PMS300) and a liquid-nitrogen-cooled InSb detector with a wavelength range of 400 nm–5 μm. The zero-order calibration was applied first to align optical paths along the devices, PMS300, and detector. Then, signal was acquired via a lock-in amplifier. A wide slit width of 2 mm was selected to collect a weak spontaneous emission signal below the *I*_th_ and a reduced width of 0.5 mm was used above *I*_th_ to detect the lasing signal with an improved spectral resolution.

For far-field diffraction pattern measurement (Supplementary Fig. S4b), the output light from InAs/InP QD laser was collected by a near-infrared objective lens (Mitutoyo 20 × Plan APO) and imaged using an infrared camera (MicroViewer 7290) with a broad spectral response ranging from 400 nm to over 2000 nm. The laser was mounted on a chip-on-carrier, and its temperature was stabilized at 20 °C using a thermoelectric temperature controller (ILK Lightwave LDT-5525B). The injection current was supplied by a Keithley 2520 current source operating in pulsed mode (1 μs pulse width, 1% duty cycle).

## Supplementary information

Supplementary information for Mid-infrared InAs/InP quantum-dot lasers

## Data Availability

The data supporting the findings of this study are available from the corresponding authors upon reasonable request.
