# Supplementary material for: Mid-infrared InAs/InP quantum-dot lasers
Source: Light Sci Appl. 2026 Jan 12;15:64. doi: 10.1038/s41377-025-02167-4 (PMC12791141; doi:10.1038/s41377-025-02167-4)
Supplement: Supplementary file 1 — Supplementary information for Mid-infrared InAs/InP quantum-dot lasers [file 41377_2025_2167_MOESM1_ESM.pdf]

Supplementary Information for

Mid-infrared InAs/InP quantum-dot lasers

Yangqian Wang[1], Hui Jia[1,*], Jae-Seong Park[1,*], Haotian Zeng[1], Igor P. Marko[2], Matthew Bentley[3], Khalil El Hajraoui[4,5], Shangfeng Liu[6], Bo Yang[7], Calum Dear[1], Mengxun Bai[1], Huiwen Deng[1], Chong Chen[1], Jiajing Yuan[1], Jun Li[1], Kongming Liu[1], Dominic A. Duffy[2], Zhao Yan[6], Zihao Wang[7], Stephen J. Sweeney[2], Qiandong Zhuang[3], Quentin M. Ramasse[4,8], Siming Chen[1], Mingchu Tang[1], Qiang Li[6], Alwyn Seeds[1], and Huiyun Liu[1]

[1]Department of Electronic and Electrical Engineering, University College London, London, UK

[2]James Watt School of Engineering, University of Glasgow, Glasgow, UK

[3]Physics Department, Lancaster University, Lancaster, UK

[4]SuperSTEM, SciTech Daresbury Science and Innovation Campus, Block J, Keckwick Lane, Daresbury, UK

[5]York NanoCentre & Department of Physics, University of York, York, UK

[6]School of Physics and Astronomy, Cardiff University, Cardiff, UK

[7]Institute of Physics, Chinese Academy of Sciences, Beijing, China

[8]School of Chemical and Process Engineering and School of Physics and Astronomy, University of Leeds, Leeds, UK

These authors contributed equally: Yangqian Wang, Hui Jia, and Jae-Seong Park

[*]Correspondence: Hui Jia (hui.jia@ucl.ac.uk) or Jae-Seong Park (jae-seong.park@ucl.ac.uk)

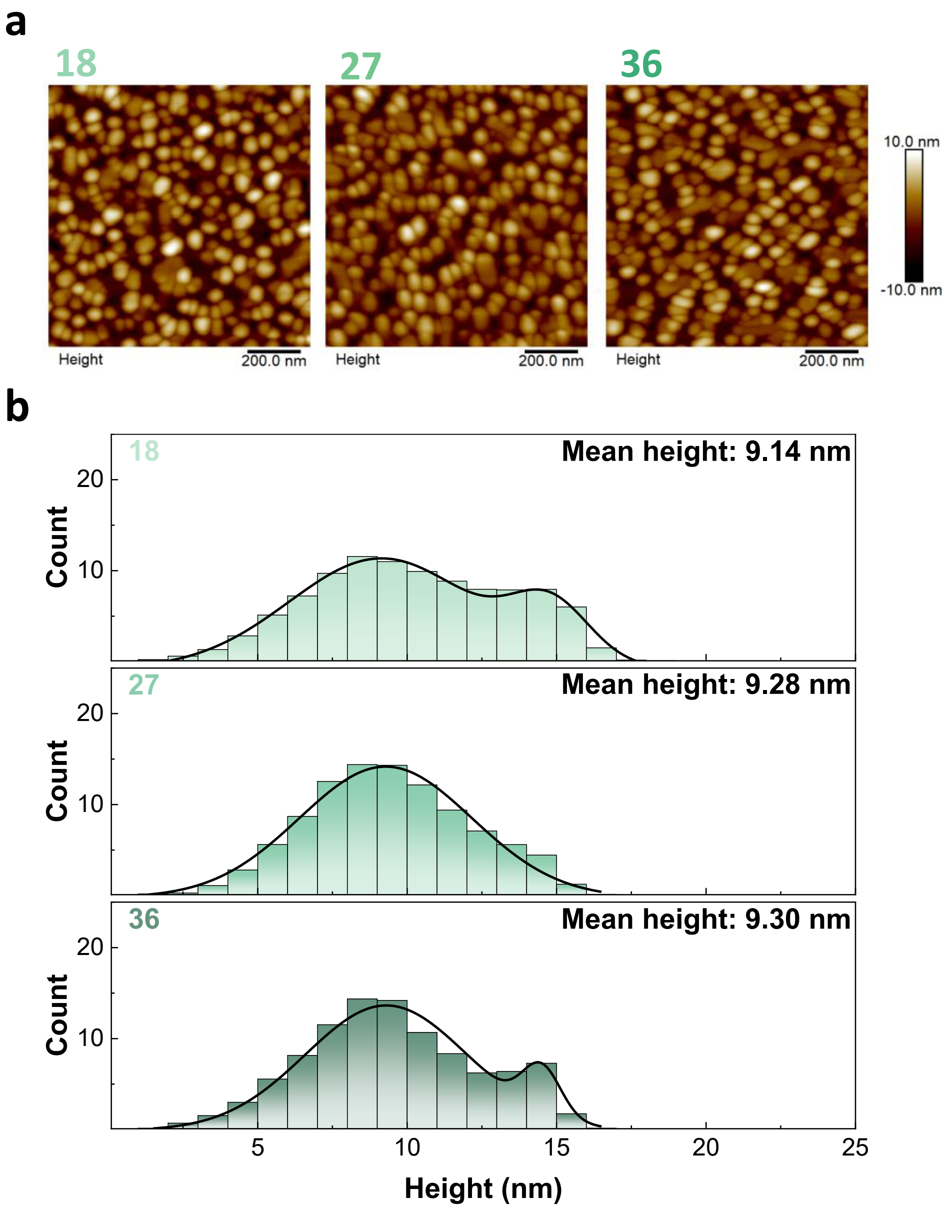

Figure S1. Optimization of dot morphology of single-layer InAs/InP QDs under different V/III ratios. a 1 × 1 μm^2 AFM scans and **b** dot-height distribution for single-layer InAs/InP QDs grown at 485 °C with 7.5 ML of InAs, under V/III ratios of 18, 27, and 36.

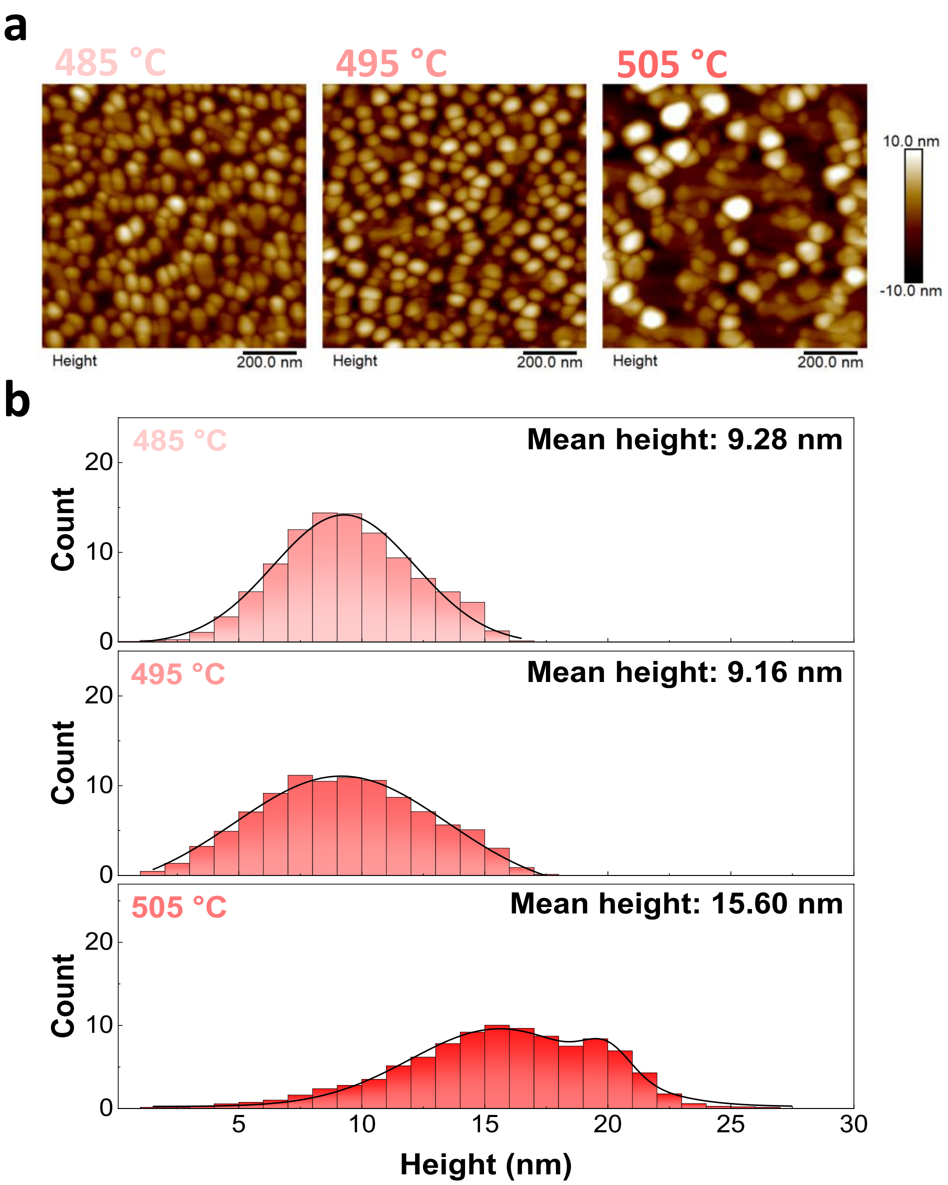

Figure S2. Optimization of dot morphology of single-layer InAs/InP QDs at different QD growth temperatures. a 1 × 1 μm^2 AFM scans and **b** dot-height distribution for single-layer InAs/InP QDs with 7.5 ML of InAs and V/III ratio of 27, grown at temperatures of 485, 495, and 505 °C.

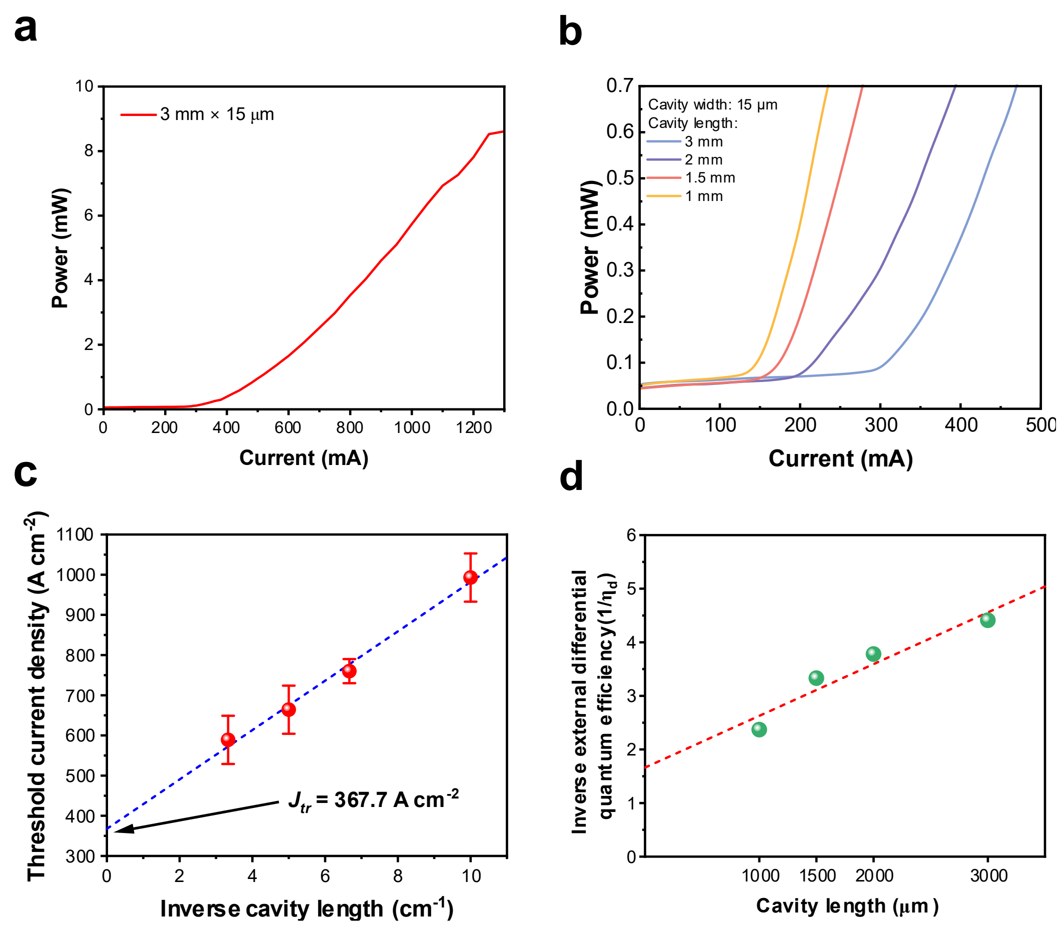

Figure S3. Laser performance characterization with varied cavity lengths. a Power versus current (L-I) curve for the 3 mm × 15 μm device at RT, showing a maximum power of 8.5 mW per facet. **b** RT L-I curves for InAs/InP QD lasers with a cavity width of 15 μm and varied cavity length from 1 to 3 mm. **c** J_{th} versus inverse cavity length for InAs/InP QD lasers, providing transparency current density (J_{tr}). The error bars indicate the total observed range (minimum to maximum) of J_{th} for all measured devices at each cavity length. **d** Inverse external differential quantum efficiency ($1/\eta_d$) versus cavity length for the InAs/InP QD lasers, where internal quantum efficiency (η_i) of 60 % and internal loss (α_i) of 6.7 cm^{-1} were calculated.

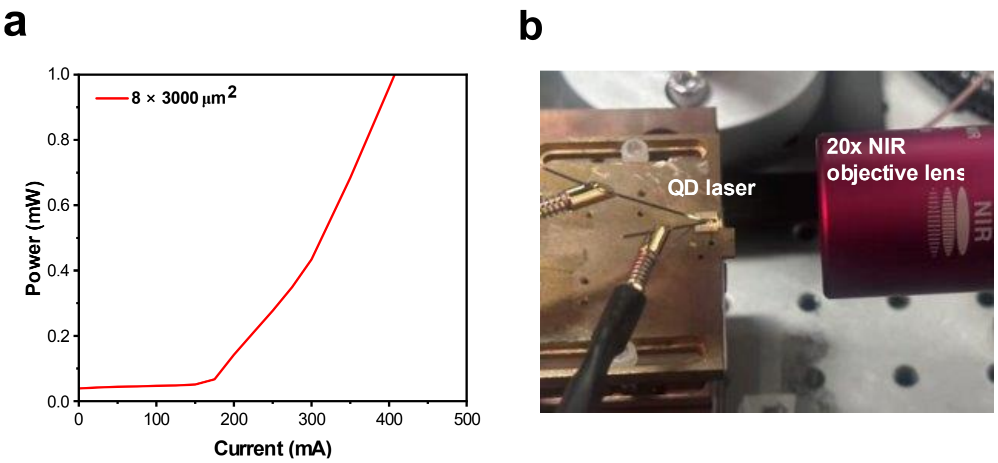

Figure S4. Far-field diffraction pattern measurement. a L-I curve at RT for the 3 mm × 8 μm device used for far-field pattern measurement, showing a I_{th} of ~ 175 mA. **b** The set-up for far-field pattern measurement.
